# Photoradical-Mediated Catalyst-Independent Protein Cross-Link with Unusual Fluorescence Properties

**DOI:** 10.1002/cbic.202300380

**Published:** 2023-08-01

**Authors:** Jinam Ravindra Bora, Radhakrishnan Mahalakshmi

**Affiliations:** a Molecular Biophysics Laboratory Department of Biological Sciences Indian Institute of Science Education and Research Bhopal Bypass Road, Bhauri, Bhopal 462066, Madhya Pradesh (India)

**Keywords:** Fluoro-indole, photo-activation, membrane protein, tyrosine oxidation, unusual fluorescence

## Abstract

Photo-actively modified natural amino acids have served as lucrative probes for precise mapping of the dynamics, interaction networks, and turnover of cytosolic proteins both *in vivo* and *ex vivo.* In our attempts to extend the utility of photo-reactive reporters to map the molecular characteristics of vital membrane proteins, we carried out site-selective incorporation of 7-fluoro-indole in the human mitochondrial outer membrane protein VDAC2 (voltage-dependent anion channel isoform 2), with the aim of generating Trp–Phe/Tyr cross-links. Prolonged irradiation at 282 nm provided us with a surprisingly unusual fluorophore that displayed sizably red-shifted excitation (λ_ex-max_= 280 nm→360 nm) and emission (λ_em-max_= 330 nm→430 nm) spectra that was reversible with organic solvents. By measuring the kinetics of the photo-activated cross-linking with a library of hVDAC2 variants, we demonstrate that formation of this unusual fluorophore is kinetically retarded, independent of tryptophan, and is site-specific. Using other membrane (Tom40 and Sam50) and cytosolic (MscR and DNA Pol I) proteins, we additionally show that formation of this fluorophore is proteinin-dependent. Our findings reveal the photoradical-mediated accumulation of reversible tyrosine cross-links, with unusual fluorescent properties. Our findings have immediate applications in protein biochemistry and UV-mediated protein aggregation and cellular damage, opening avenues for formulating therapeutics that prolong cell viability in humans.

## Introduction

Membrane proteins are fundamental components of cellular life, constituting one-third of the entire proteome of any cell. These proteins perform diverse and essential functions from metabolite transport, signaling, and regulation, to timed cellular apoptosis,^[1]^ making them indispensable for the proper functioning of every cell. Structurally, membrane proteins are classified as either transmembrane helices or β-barrels.^[2]^ Only three transmembrane β-barrels are known to exist in humans, found exclusively in the outer membrane of mitochondria,^[3]^ and critical for cell survival. However, the physico-chemical characteristics and structural dynamics of membrane β-barrels are deduced largely from proteins of bacteria and lower eukaryotes.^[3b,4]^ This is due to significant challenges in producing folded β-barrel samples of sufficient stability for biochemical characterization, and the limited availability of molecular reporters of elements essential for their regulation.^[1f,3b,4f,5]^ Therefore, we need alternative approaches for direct biophysical characterization of mitochondrial β-barrels.

The voltage-dependent anion channel (VDAC) is one of the three mitochondrial β-barrel outer membrane proteins (mOMPs), and is also the most abundant.^[6]^ It exists as three nearly-identical isoforms in humans (hVDAC1, 2, 3), and under physiological conditions, all three isoforms transport metabolites and regulate cellular ATP supply.^[6b,7]^ Additionally, VDACs play essential auxiliary roles in mitochondrial protein import, steroidogenesis, Ca^2+^ homeostasis, and redox regulation.^[1d,8]^ Of particular therapeutic interest is the human VDAC2 (hVDAC2) isoform.^[6a,b,9]^ Since hVDAC2 promotes cancer cell survival, small molecule inhibitors that disrupt hVDAC2 function are being explored for cancer therapy.^[10]^ hVDAC2 is also a promising candidate for neurodegenerative diseases, including heart failure,^[9b,11]^ amyotrophic lateral sclerosis,^[12]^ hypoxia,^[13]^ epilepsy,^[14]^ Parkinson’s^[15]^ and Alzheimer’s disease.^[16]^ However, our limited knowledge of this specific isoform has severely hindered the design and development of peptidomimetics and small molecule inhibitors for VDAC2 functional modulation. Therefore, gaining a better molecular understanding of hVDAC2 structures, functional dynamics, and potential regulatory sites, is needed urgently.

Environment-sensitive fluorophores, particularly tryptophan residues, serve as excellent reporters of local dynamics, biomolecule stability, and molecular interaction networks,^[17]^ and have been used extensively to characterize proteins. Indeed, precise spatial positioning of Trp can result in a Trp–Phe fluorophore, as seen in photo-activated cyclophilin A (CypA).^[18]^ In turn, the formation of such hyperfluorescent structures serves as reporters of explicit aromatic interaction geometries and the dynamics of such interactions. The four lipid-facing intrinsic interface tryptophans (W75, W86, W160, W221) of hVDAC2 differentially regulate β-barrel formation and post-folding stability of this β-barrel.^[19]^ Hence, we sought to deduce the local dynamics and strength of the interface aromatic interaction network of hVDAC2 through photo-activated cross-linking of selectively positioned 7-fluoro–L-tryptophan (7F–^L^Trp)’ Unexpectedly, we found the formation of a fluorescent entity with unique excitation and emission maxima, independent of 7F–^L^Trp. Additionally, we find that treatment with organic solvents can readily reverse this modification. By extending our studies to the two other human mitochondrial outer membrane proteins Tom40 and Sam50,^[5a]^ and two soluble prokaryotic proteins (bacteriophage DNA polymerase and *Mycobacterium* MscR),^[20]^ we propose the involvement of tyrosine oxidation^[21]^ in forming this unique fluorophore. Further investigation of the molecular structures of this unusually fluorescent and reversible aryl modification will have direct applications as probes for understanding protein folding, function, and protein–protein interactions, using fluorescence based studies. Our studies also implicate tyrosine modifications as a leading cause of UV-induced protein oxidation, particularly in those cells facing direct UV exposure (e.g., epithelial (skin) cells, retinal and corneal cells in the eye), emphasizing the immediate need for therapeutic interventions in preventing irreversible protein damage.

## Materials and Methods

### Mutagenesis and Trp-library preparation

pET3a vector carrying the full-length human *VDAC2* gene codon-optimized for expression in *Escherichia coli* was generated with a C-terminal His_6_-tag and a Gly linker.^[22]^ The single-Trp variants were generated by replacing the three other intrinsic Trp codons with Phe, as reported earlier.^[19]^ Additionally, Trp residues were introduced in non-canonical sites using the Trp-less VDAC2 gene (pET3a–*VDAC2*^W–^) as the template, through site-directed mutagenesis.^[19]^ The mutation in each case was confirmed using Sanger sequencing.

### Protein expression as inclusion bodies

All buffers and reagents for protein expression and purification were obtained from Sigma-Aldrich Chemicals Pvt. Ltd., Merck & Co., Inc., HiMedia Laboratories Pvt. Ltd., or MP Biomedicals, Inc.. hVDAC2 WT, W0, and the single tryptophan mutants were overexpressed as inclusion bodies in *E. coli* C41 cells. The cells were cultured in minimal media (M9) containing 10 g/L D-glucose as the primary carbon source, a cocktail of all 20 amino acids (500 mg/L each of Ser, Asp, Glu, Phe and Tyr; 300 mg/L of Met, Gly and Cys; 200 mg/L of all others), 50 mM Na_2_HPO_4_, 22 mM KH_2_PO_4_, 8.5 mM NaCl, 0.1 M MgSO_4_, 0.01 M CaCl_2_, 100 mg/L thiamine HCl, and 100 mg/L ampicillin.^[23]^ For incorporation of 7-fluoro-L-tryptophan (7F–^L^Trp), 25 mg/L 7-fluoro-indole was added and tryptophan was omitted from the amino acid cocktail. *E. coli* C41 cells were first grown in 5 ml Luria-Bertini broth for 5 h at 37°C. 1 ml of this culture was transferred to 100 ml M9 medium (containing all 20 amino acids) and allowed to grow at 37°C for 12 h. This overnight culture was transferred to a 1 L M9 medium, and incubated at 37°C till the OD_600_ reached 0.8. Cells were pelleted by centrifugation at 4 °C, 6,150 x*g*, for 15 min. The cell pellet was re-suspended in M9 medium (without Trp, with 7-fluoro-indole) and protein production was induced by addition of 1 mM isopropyl β-D-1-thiogalactopyranoside (final concentration) for 5 h at 37°C. Cells were then harvested by centrifugation at 4°C, at 8,850 x*g*, and stored at –86°C until further processing. When no fluoro-substitution was needed, cells were directly induced when the OD_600_ = 0.8 was achieved.

### Protein extraction and purification

To extract the protein from inclusion bodies,^[5c,22]^ the cell pellet was first re-suspended in 20 mM Tris-HCl pH 8.0. Cell membranes were disrupted by incubation with 12.5 μg/mL lysozyme for 20 min, followed by probe sonication for 10 min. The sonicated sample was added to a 2.75% solution of Triton X-100, incubated at 37°C for 20 min, and then subjected to centrifugation at 10,500 x *g*. The pellet was then washed with 0.5% sodium deoxycholate containing 5 mM dithiothreitol (DTT), followed by 2 mM CaCl_2_ treatment. Finally, the pellet was re-suspended in methanol, incubated for 10 min at 37°C, at 200 rpm, and the protein pellet was recovered by high-speed centrifugation at 4°C, 46,750 x *g*. The protein pellet was lyophilized to remove trace water and methanol, and stored at - 86 °C.

The protein pellet was dissolved in binding buffer (6 M GdnHCl, 50 mM phosphate buffer pH 7.2, 15 mM imidazole, 10 mM DTT), by heating at 60°C for 15 min. The undissolved components were removed by centrifugation at 4°C at 19,000 x*g*, and the supernatant collected was diluted with binding buffer such that it had 1–2 mg/ml of protein and at least 2 mM DTT. The sample was applied on a Ni-NTA HisTrap FF column (GE Healthcare Pvt. Ltd.) using the EP-1 Econo pump (Bio-Rad Laboratories, Inc.). The column was washed with 7 column volumes of the binding buffer. Samples were eluted using 4 column volumes of the elution buffer (binding buffer with 400 mM imidazole and 2 mM DTT). Eluted fractions containing only VDAC2 (without co-elulting impurities) were pooled, supplemented with ethylenediaminetetraacetic acid (EDTA; 2.5 mM final concentration), and dialyzed against a 250-fold excess of milli-Q for at least 48 h (10–12 changes) in a 1 kD cut-off Spectra/ Por dialysis membrane, and stored in -86 °C as a lyophilized powder.

### VDAC2 folding

hVDAC2 protein powder was dissolved in 50 mM phosphate buffer pH 7.2 containing 100 mM NaCl, 6 M GdnHCl, 10 mM DTT by heating at 60°C for 10 min, and centrifuged at 4°C at 19,000 x*g* to remove undissolved constituents. The supernatant was quantified to obtain a concentration of 250 μM protein, using the molar extinction coefficient of 36,900 M^−1^ cm^−1^ for hVDAC2-WT, 20,400 M^−1^ cm^−1^ for the single-Trp variants, and 14,900 M^−1^ cm^−1^ for W0.^[22]^ Extinction coefficients were obtained using the ProtoParam tool (Expasy, Swiss Institute of Bioinformatics). An impurity level of 10% was considered for quantifying preparations directly from inclusion bodies. The extinction coefficient of phenylalanine was used in place of 7F–^L^Trp. The unfolded stock was diluted 10-fold with buffer-detergent mixtures to final concentrations of 25 μM protein, 19.5 mM *n*-dodecyl β-D-maltoside (DDM), 0.6 M GdnHCl, 50 mM phosphate buffer pH 7.2, 100 mM NaCl, and 10 mM DTT. The reaction was incubated for 12 h at 4°C with rotational mixing. The folded stock was centrifuged at 19,000 x*g* at 4°C to remove insoluble aggregates, and the supernatant was re-quantified.

### CD spectropolarimetry and thermal denaturation experiments

The folded samples were diluted 2-fold using folding buffer into a 500 μL cuvette with 1 mm path length (Hellma Analytics, Germany),such that concentrations of all constituents except protein and GdnHCl were maintained. Both wavelength scans and thermal denaturation measurements were monitored using far-UV circular dichroism (CD) spectropolarimetry on a JASCO J-815 instrument (JASCO Corp., Japan). For thermal denaturation experiments, the raw ellipticity (in mdeg) at 215 nm and 222 nm were measured from 4–95 °C with a ramp rate of 1 °C/min, and 92 data points were collected for each wavelength.^[22a,24]^ The temperature was maintained within +/–0.1 °C of the target temperature for 5 s, followed by data acquisition. All measurements were done with a bandwidth of 1 nm with a data integration time of 1 s. The temperature was restored to 4°C after completion of the unfolding experiment. CD wavelength scans were recorded pre- and post-melting from 210–260 nm with a scanning speed of 50 nm/min and a data interval of 0.5 nm. Three accumulations were done for each sample to improve the signal-to-noise ratio, and spectra were averaged, corrected for buffer and background contributions, smoothened, converted to molar ellipticity, and plotted.

### UV cross-linking experiments

Cross-linking of samples was carried out by irradiating the folded protein. For this, sample preparations (200 μL or 2.5 mL) were aliquoted into quartz cuvettes (path lengths of 10 mm x 2 mm or 10 mm x 10 mm, respectively) and irradiated at 25 °C using a 150 W Xe lamp with an excitation wavelength (λ_ex_)=282 nm on a FluoroMax-4 spectrofluorometer (Horiba Scientific Inc.). For larger volumes, samples were additionally stirred constantly during irradiation. Cross-linking kinetics were recorded with time at an emission wavelength (λ_em_)= 430 nm. The excitation slit width was kept at 16 nm, while emission slit width was set at 5 nm for smaller volumes and 2 nm for larger volumes. The kinetics were recorded for 25 min (smaller volumes) or 250 min (larger volumes; 60,000 data points). Data were fitted to an exponential rise to maximum function using SigmaPlot v14 (Systat Software), and results from 2 independent experiments were used to calculate the standard deviation.

### Steady-state fluorescence spectroscopy

Steady-state scans were also recorded on the FluoroMax-4 (Horiba Scientific Inc.). Excitation scans were done using λ_ex_=250–400 nm with a fixed λ_em_= 430 nm. Emission scans were obtained using λ_ex-max_= 280 nm with λ_em_= 300–500 nm, and λ_ex_=360 nm (or the respective excitation maxima for the mutant) with λ_em_= 390–600 nm. Data were acquired with a 1 nm increment, and corrected for background contributions. All scans were done at room temperature (25 °C), with a 5 nm excitation slit width, and 5 nm emission slit width for smaller volumes and 3 nm emission slit width for larger volumes.

## Results

### Incorporation of 7-fluoro–L-tryptophan in hVDAC2

**Figure 1:**
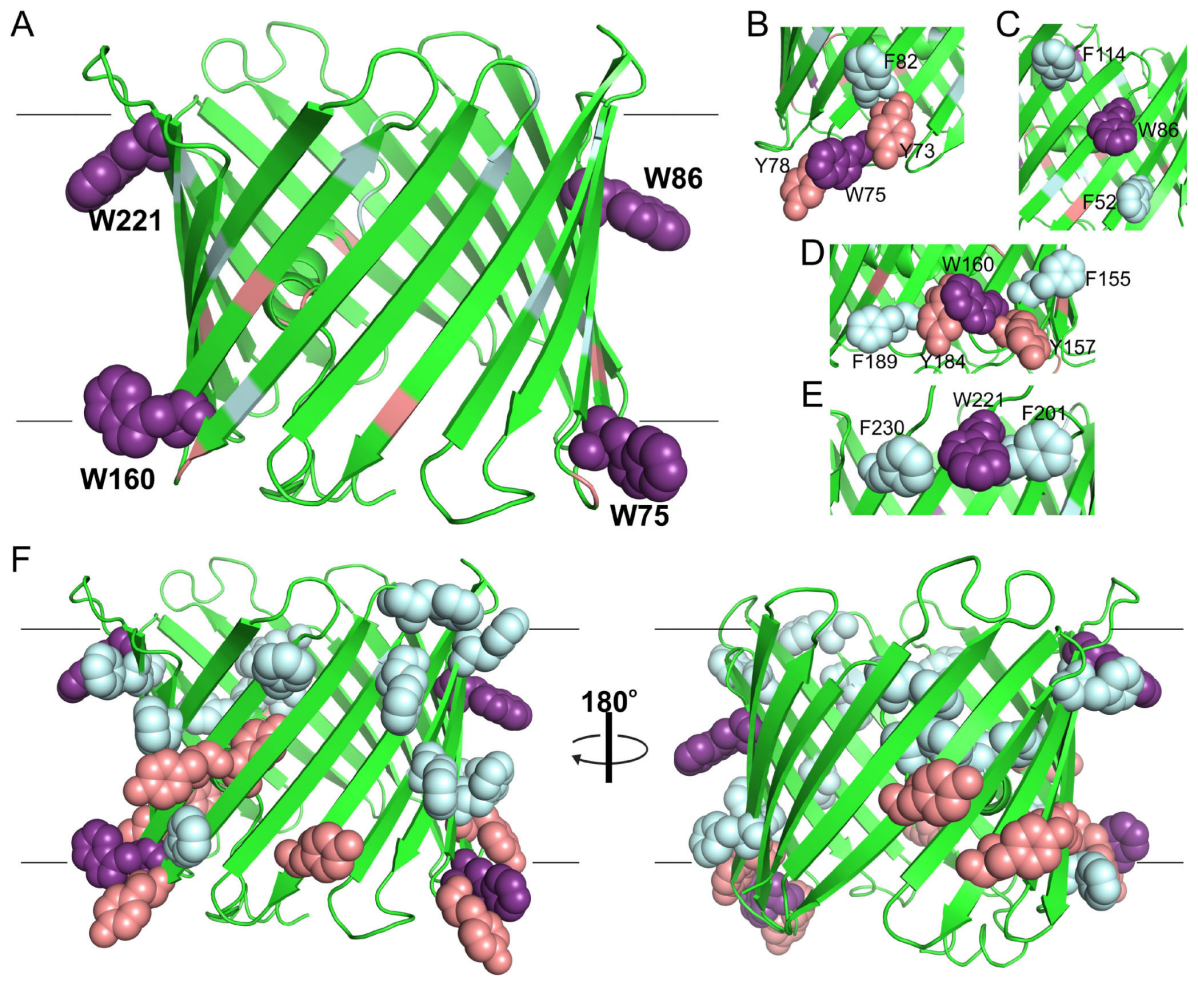
I-TASSER modeled structure of human VDAC2.^[19]^ (A) Cartoon representation of the β-barrel highlighting the four interface Trp residues (dark violet) as space-filling model. Each indole, namely, W75 (B), W86 (C), W160 (D), and W221 (E), establishes a unique interaction environment with spatially proximal Phe (pale cyan color) and Tyr (salmon color). Note that W86 possesses aryl residues only in its 10 Å vicinity. (F) Structure of the complete barrel highlighting all three aryl groups. Position of the bilayer is marked in both (A) and (F) with straight lines.

The human VDAC2 has four intrinsic Trp residues, namely, W75, W86, W160, and W221. All four tryptophans are located at the membrane interface, wherein the indole side chains pointing towards the lipid headgroups and form both hydrophobic and electrostatic interactions (Figure 1). In the folded hVDAC2 β-barrel structure, each indole possesses a unique interaction environment with spatially proximal aliphatic and aromatic side chains. For example, W75 is flanked by two phenyl rings of vicinal Tyr residues with which it forms C–H…π (edge-to-face) interactions, with one distal Phe. Similarly, W160 establishes interactions with two proximal Tyr (C–H…π geometry) and two distal Phe. W221 is sandwiched between two benzyl rings in C–H…π geometry, with no vicinal Tyr. The W86 indole does not form aromatic interactions, as it is surrounded exclusively by aliphatic groups. Hence, we reasoned that if these interactions are rigid in hVDAC2, by incorporating a singly fluorinated indole in at least three of the four positions can photocatalytically form Trp–Phe/Tyr cross-links.

We generated single-Trp variants of hVDAC2,^[19]^ and selectively incorporated a fluorine atom in place of H^ζ2^ of the indole ring. We achieved this by growing *E. coli* (C41) cells in minimal media (depleted of nitrogen salts) supplemented with 7-fluoro–indole (in place of Trp). In addition, a Trp-less hVDAC2 variant (W0) was also generated as control for structural studies. We reconstituted all the proteins by folding in *n*-dodecyl β-D-maltoside (DDM),^[22b]^ and assessed the efficiency of folding by monitoring the aggregation using high-speed centrifugation of all samples. We observed that all proteins could be folded successfully in DDM at final concentrations of 25 μM protein and 19.5 mM DDM.

**Figure 2:**
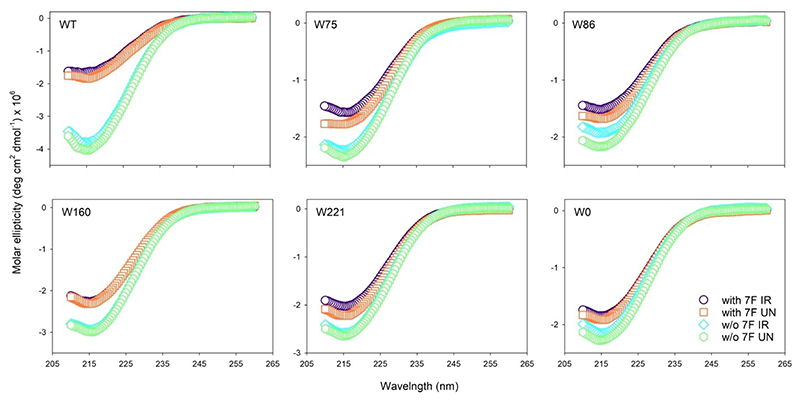
Far-UV CD wavelength scans of folded hVDAC2 variants with(out) 7-fluoro–indole. Note how the secondary structure content is substantially lowered when 7F–Trp is incorporated in WT, with the spectra matching the single-Trp variants and hVDAC2–W0. Hence, presence of an electronegative group at the ζ2 position lowers the local indole hydrophobicity at the bilayer interface, and affects the secondary structure content of hVDAC2 folded in DDM. However, sample irradiation (IR: irradiated with λ_ex_=282 nm for 25 min; UN: not irradiated) does not alter the protein structure.

Being an electron withdrawing group, adding fluorine to the indole ring of tryptophan can lead to lowered overall folding efficiency of hVDAC2 in DDM. Indeed, we have previously shown the importance of tryptophans in hVDAC2 structure and stability.^[19]^ Therefore, to assess if substitution of the intrinsic Trp residues, and the additional incorporation of 7F–^L^Trp affected hVDAC2, we carried out far-UV circular dichroism analysis. Comparison of the molar ellipticity values of folded hVDAC2 variants (WT, single-W, W0) (Figure 2) shows that the substitution of Trp residues influences the β-sheet content of this protein in a site-specific manner, affecting each mutant differently. The hVDAC2–W86 variant shows the lowest secondary structure content (Figure 2). The most significant effect is seen in WT, when all four indoles are substituted, suggesting that altering the local electrostatics with fluorine affects the structure of the hVDAC2 barrel in a manner similar to mutating the residue (compare WT–7F with W0 in Figure 2). Overall, we find that any change to the intrinsic Trp residues (particularly 75, 160, and 221) can alter the structure of hVDAC2 folded in DDM, and is in line with our previous findings.^[19]^

**Figure 3:**
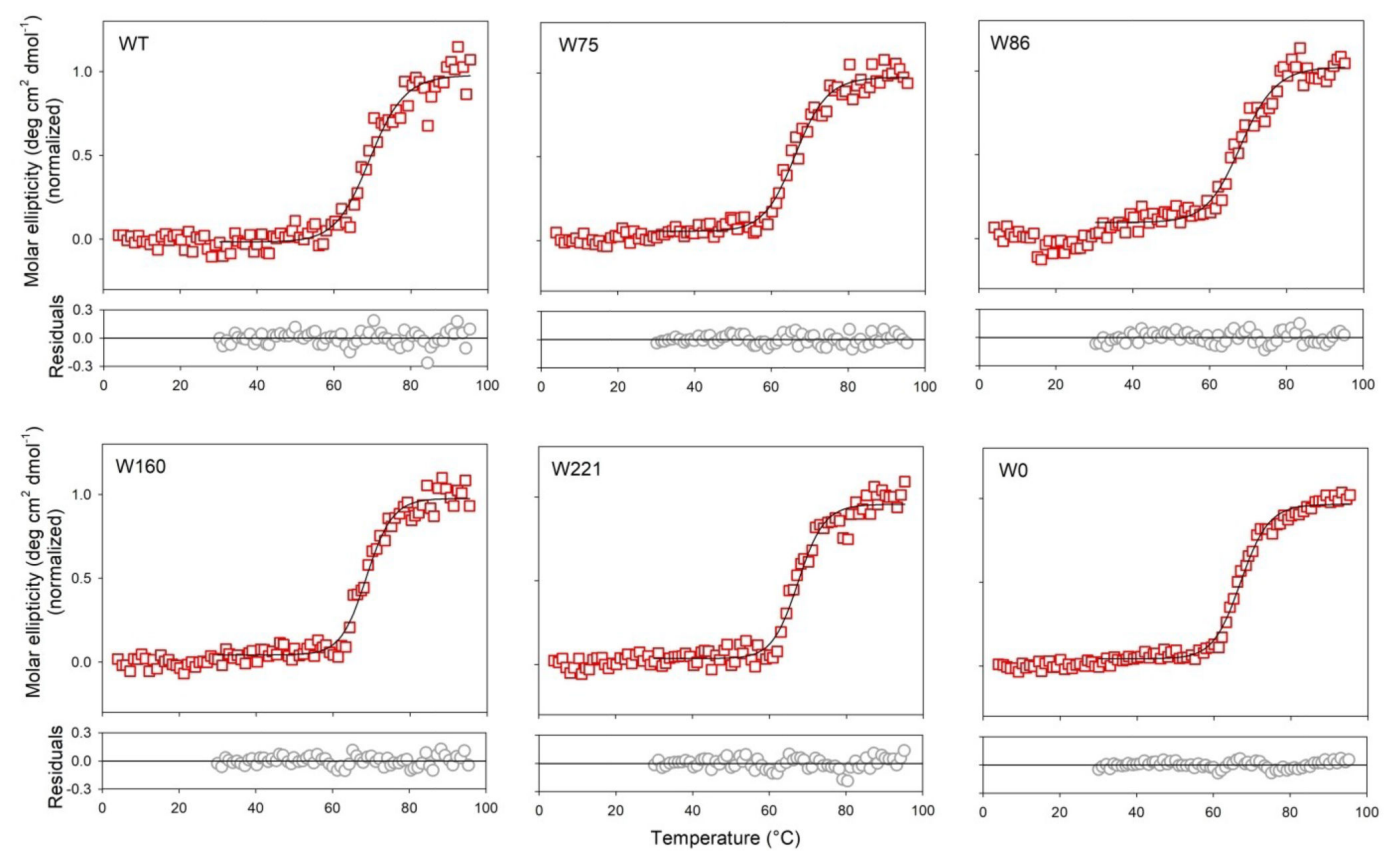
Thermal denaturation experiments of hVDAC2 containing 7F–^L^Trp pre-irradiation. Shown are the unfolding profiles monitored at θ_215_ using far-UV CD. Data from 30-95 °C were fitted to a two-state thermal unfolding model to obtain the mid-point of thermal denaturation (T_m_). The data is normalized by assigning the mean of ~15 initial points to 0 and the last ~10 points to 1. Fits are shown as black sold lines and the residuals are provided below each graph. Thermal denaturation data for un-irradiated proteins containing ^L^Trp are shown in Figure S2. Also see Figure S3 for far-UV CD wavelength scans recorded at the end of the unfolding experiment.

**Figure 4:**
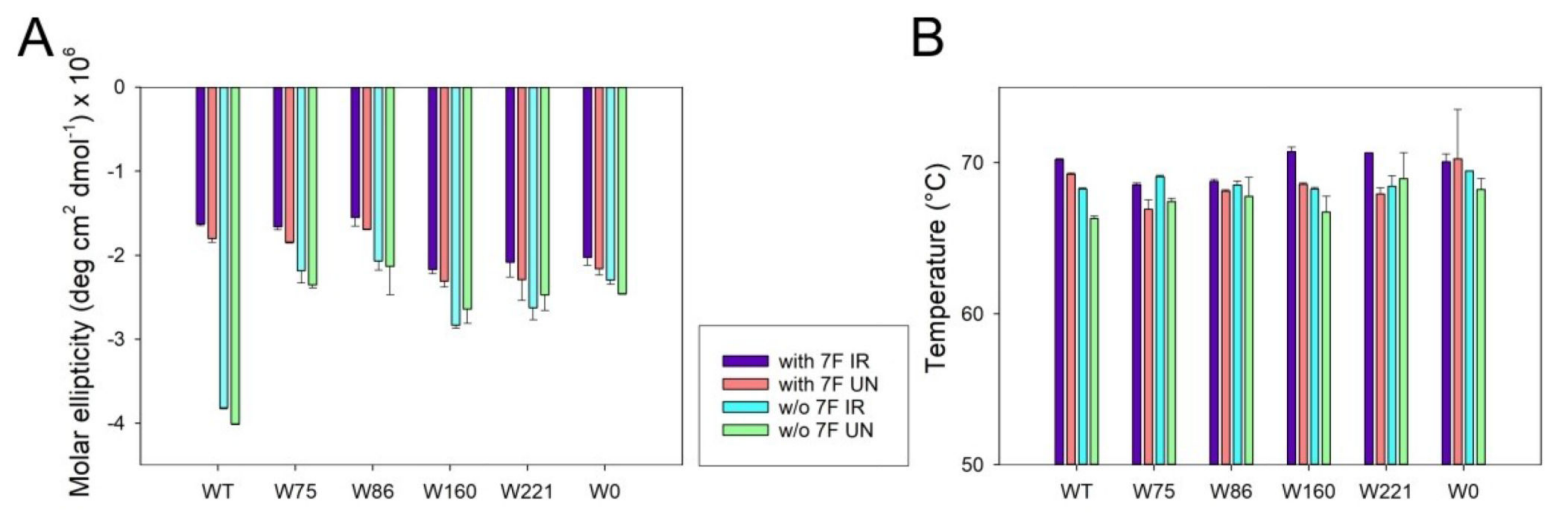
Histogram comparing the secondary structure content (θ_215_) (A) and thermal denaturation (T_m_) mid-point (B) across hVDAC2 variants. Shown are results obtained for proteins with or without (w/o) 7F–^L^Trp (7F) and either not irradiated (UN) or exposed to 282 nm irradiation (IR). Error bars were calculated from two independent experiments, and the complete data are provided in Table S1. Note that the θ_215_ (with the exception of WT containing ^L^Trp) and T_m_ are independent of 7F–^L^Trp and irradiation.

To check if hVDAC2 folded in DDM is indeed stable, we additionally performed thermal denaturation measurements by monitoring the change in secondary structure content at θ_215_ (Figures 3, S2, S3). Fitting the denaturation curves to a two-state thermal unfolding model provided us with T_m_ values (mid-point of thermal denaturation) that were comparable across all constructs, irrespective of fluorine incorporation (Figure 4, Table S1). This showed that the proteins were stably folded, and that effect of fluorine on the barrel’s stability is not as significant as its effect on the secondary structure content. In addition, chemical denaturation experiments obtained by monitoring the fluorescence of the intrinsic Trp (or 7F–^L^Trp), to derive the dependence of the folded protein on the denaturant concentration showed no difference between the hVDAC2 Trp variants with(out) 7F–^L^Trp (data not shown). Put together, we deduce that the incorporation of 7-fluoro-indole does not substantially alter the ability of hVDAC2 to fold correctly in DDM, and supports the formation of a structurally stable β-barrel in solution.

### Photo-activated hVDAC2 variants show unusual fluorescence

**Figure 5:**
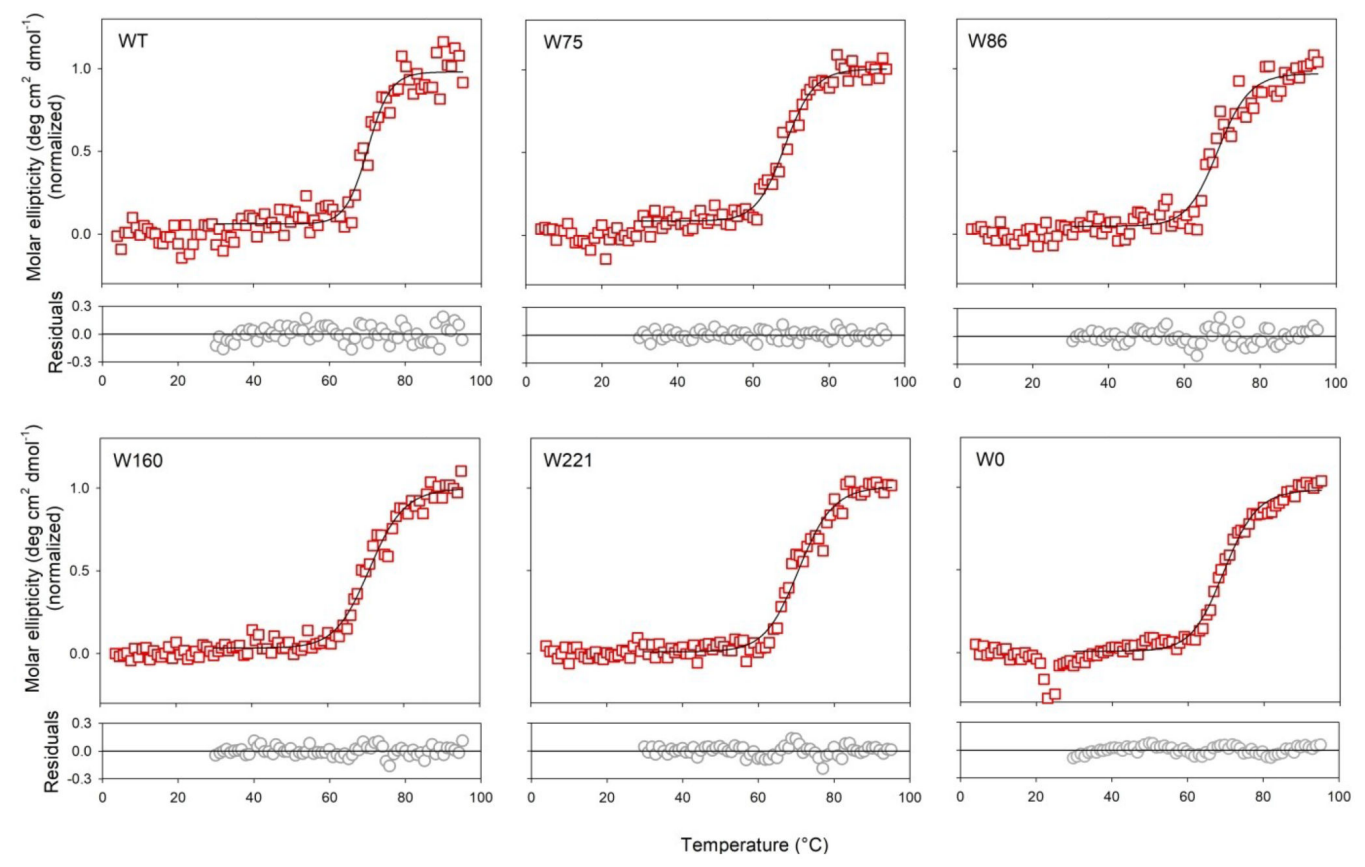
Thermal denaturation experiments of post-irradiated 7F–^L^Trp-containing hVDAC2 variants folded in DDM. Shown are the unfolding profiles monitored at θ_215_ using far-UV CD. Data from 30–95 °C were fitted to a two-state thermal unfolding model to obtain the mid-point of thermal denaturation (T_m_). The data is normalized by assigning the mean of ~15 initial points to 0 and the last ~10 points to 1. Fits are shown as black sold lines and the residuals are provided below each graph. Also see Figure S4 for thermal denaturation data for irradiated proteins containing ^L^Trp.

As shown with CypA,^[18]^ 7F–^L^Trp undergoes photo-activated cross-linking with neighboring aryl rings, when irradiated with 282 nm.^[18]^ The resultant formation of a hyperfluorescent structure provides a direct read-out of stereo-specific interactions between Trp and Phe/Tyr. Accordingly, we subjected hVDAC2–WT (WT–7F) and the single-Trp variants (W75–7F, W86–7F, W160–7F, and W221–7F), pre-folded in DDM, to UV irradiation with λ_ex_= 282 nm, using a 150 W Xe lamp. We tested the secondary structure content of the post-irradiated samples using far-UV CD wavelength scans (Figure 2), and monitored the stability of these samples using thermal denaturation and measurement of the T_m_ (Figures 4, 5, S4, Table S1). Comparison of the CD spectra and θ_215_ for all hVDAC2 variants with(out) 7F–^L^Trp and post-irradiation showed us no significant change in the structure or stability of these proteins (Figure 4). Hence, irradiation does not adversely affect hVDAC2 stability.

**Figure 6:**
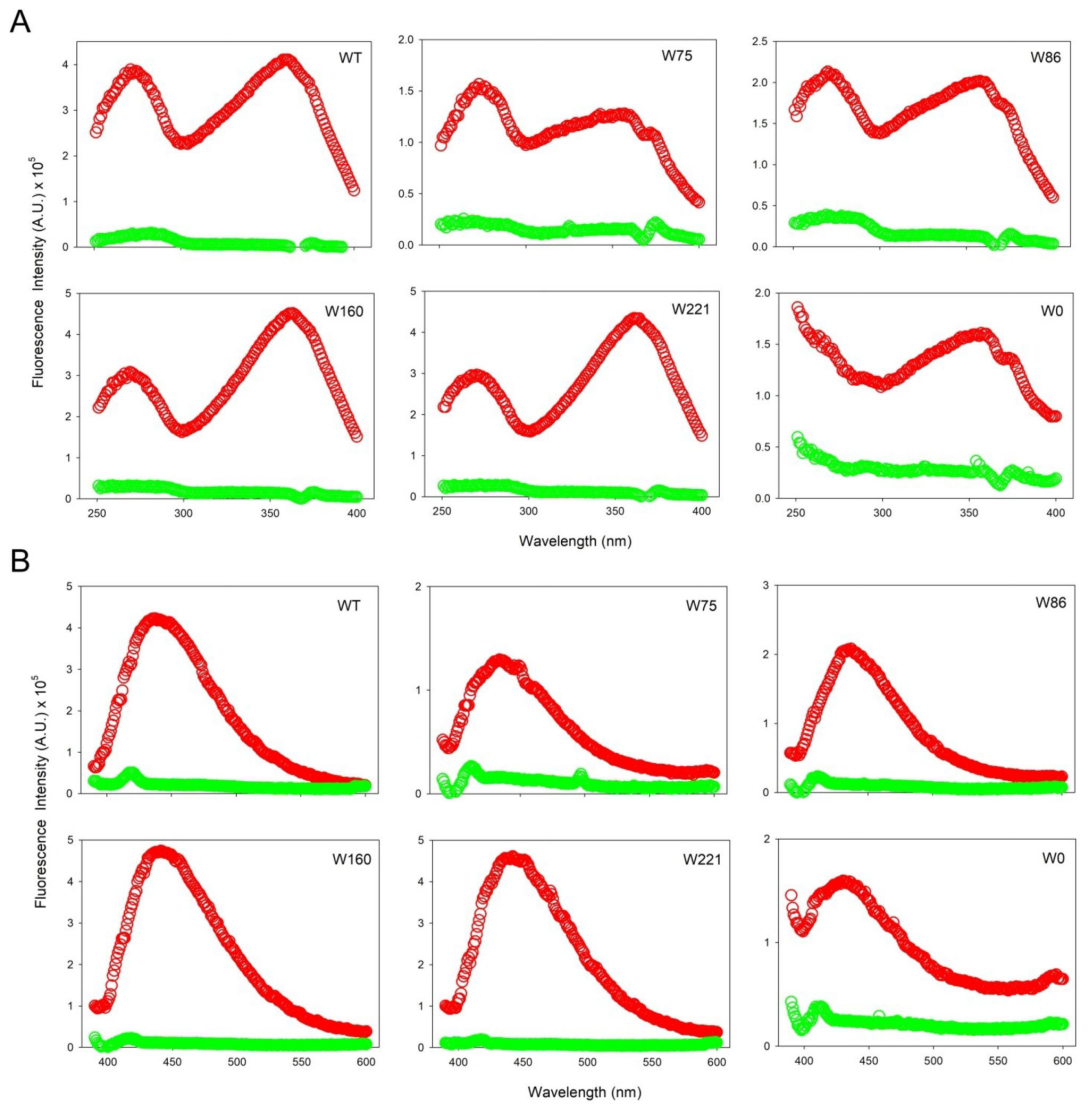
hVDAC2 shows unusual florescence post-irradiation. Excitation (A) and emission (B) scans of hVDAC2 variants containing 7F–^L^Trp exhibit a substantially blue-shifted λ_ex-max_× of 330 nm and a corresponding emission spectrum with a λ_em-max_=430 nm. Spectra recorded pre-irradiation are in green, and post-irradiation are in red. Note that W0 (with no tryptophan) also shows a similar red-shifted fluorescence post-irradiation, suggesting the involvement of other aryl residues in formation of the unusual fluorophore. Also see Figure S5 for emission spectra recorded using λ_ex_= 280 nm.

**Figure 7:**
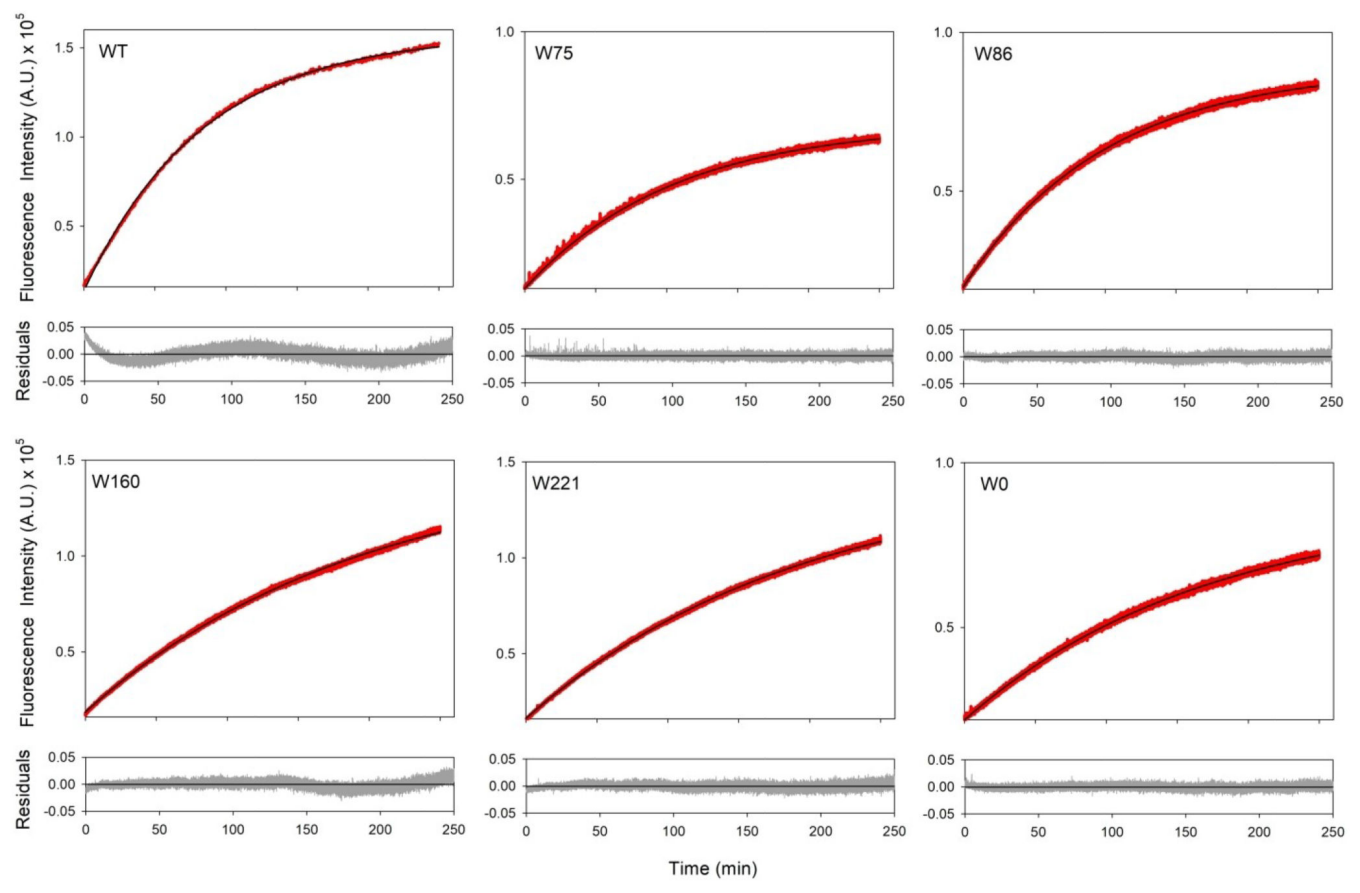
UV irradiation and cross-linking kinetics. Folded hVDAC2 W0 and variants containing 7F–^L^Trp, were irradiated at 282 nm and the kinetics of the reaction was monitored using fluorescence emission at 430 nm. The data was fitted to a single exponential function (data are shown in red, fits as solid black lines, and residuals are shown in green in the plot below), and the rates were deduced. Note how the kinetics of the process is faster in WT, W75 and W86, suggesting that the rates do not depend on the number of tryptophans, but on their local environment. Also see Figure S6 for data with other hVDAC2 variants.

Interestingly, UV irradiation showed a remarkable increase in the sample fluorescence, which was accompanied by a significant red shifted excitation spectrum (change in the λ_ex-max_ from 280 nm to 360 nm; Figure 6A) and emission spectrum (change in λ_em-max_ from 330 nm to 430 nm; Figure 6B). We obtained similar results for all hVDAC2 variants (WT and all single-Trp) carrying 7F–^L^Trp (Figures 6, S5), indicating that formation of the modified fluorophore was independent of the vicinal aryl residues (note that W86 is flanked by only aliphatic residues; see Figure 1). Put simply, all end-point samples showed red-shifted excitation and emission spectra. Hence, to probe the rate of photo-activated modification of 7F–Trp, we monitored the kinetics of the reaction at 430 nm for 250 min, with constant stirring. The time-dependent fluorescence increase we observed (Figure 7) were fitted to a single exponential function, providing the rate constants listed in Table 1. Surprisingly, for all the hVDAC2 variants, the cross-linking rate constants we obtained are 10-fold lower than what was observed for CypA.^[18]^ An additional difference we obtained is the substantially red shifted emission with λ_em-max_ = 430 nm, whereas cross-linked CypA exhibited a λ_em-max_= 373 nm.^[18]^ To assess the position-specific effect on formation of this unusual fluorophore, we generated three additional hVDAC2 single-Trp variants containing 7F–^L^Trp and irradiated folded proteins (Figure S6). Our results confirmed the formation of the unusual fluorophore in all three proteins.

**Figure 8:**
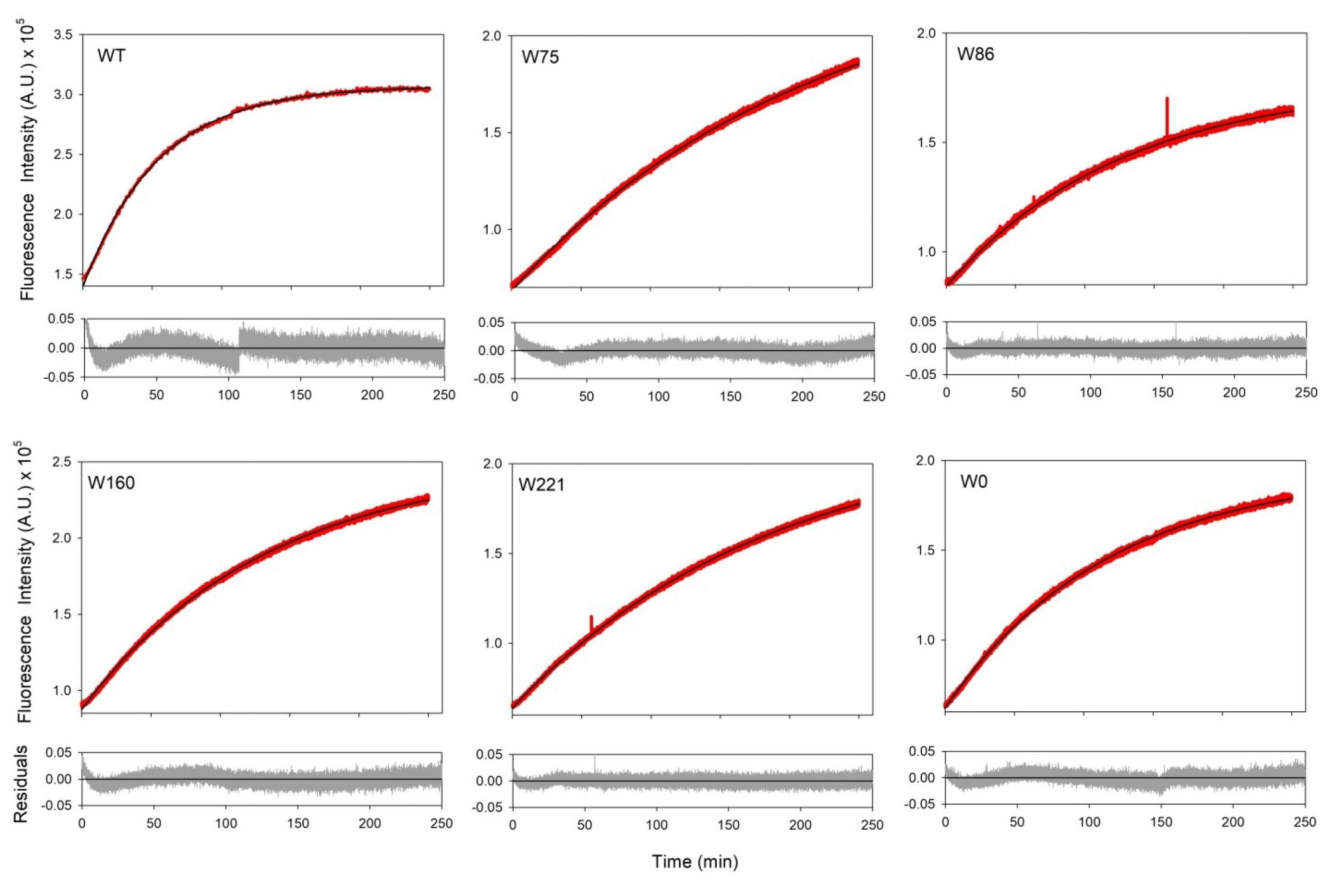
UV irradiation and cross-linking kinetics. Folded hVDAC2 W0 and variants containing ^L^Trp, were irradiated at 282 nm and the kinetics of the reaction was monitored using fluorescence emission at 430 nm. The data was fitted to a single exponential function (data are shown in red, fits as solid black lines, and residuals are shown in green in the plot below), and the rates were deduced. Note how we observe cross-linking kinetics even in the absence of 7F–^L^Trp and in W0, suggesting that the unusual modification occurs at another aryl residue, possibly tyrosine.

**Figure 9:**
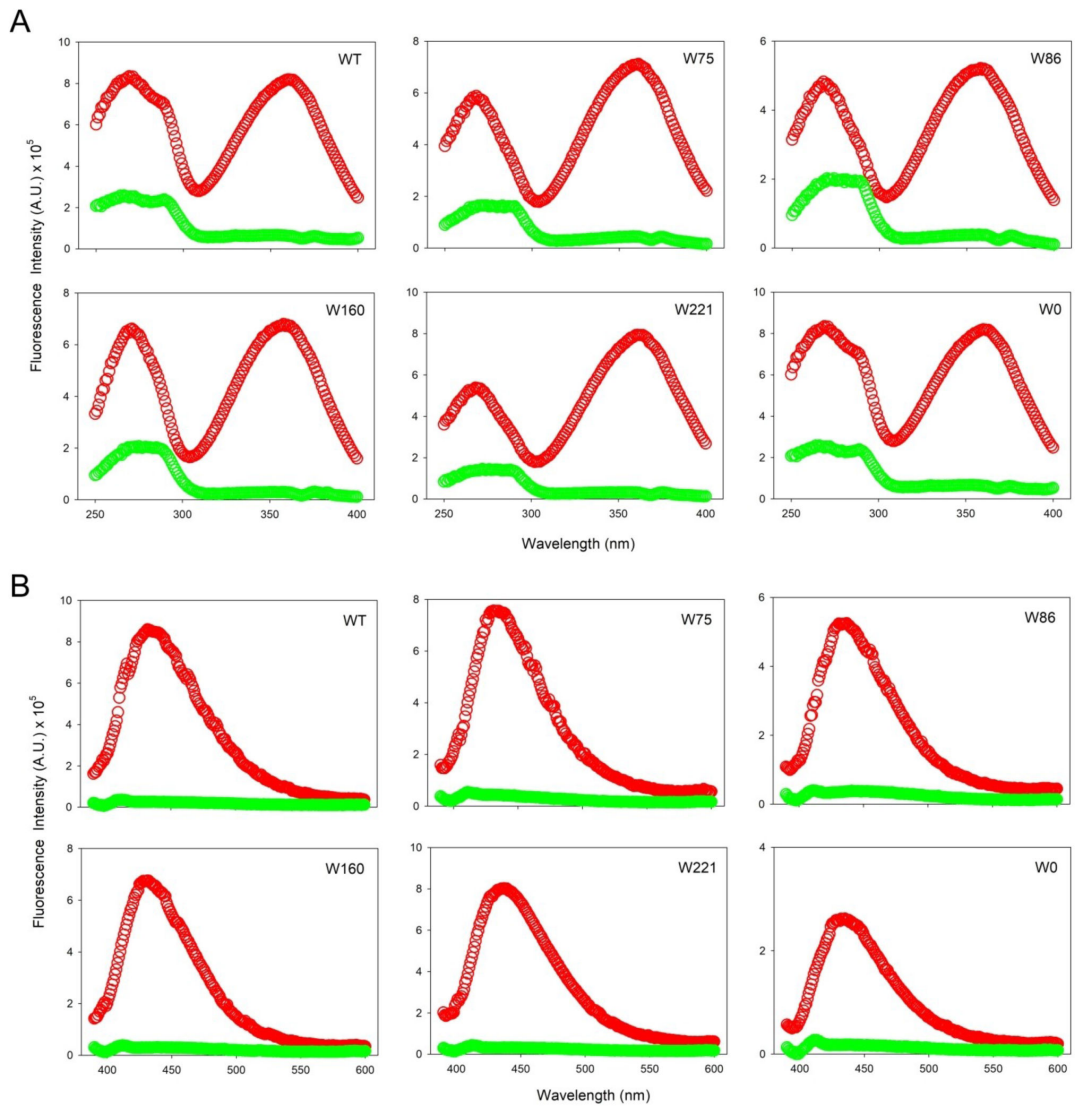
hVDAC2 shows unusual florescence post-irradiation irrespective of 7F–^L^Trp. Excitation (A) and emission (B) scans of hVDAC2 variants containing ^L^Trp when subjected to irradiation at 282 nm for 250 min exhibit a substantially blue-shifted λ_ex-max_ of 330 nm and a corresponding emission spectrum with a λ_em-max_=430 nm. Spectra recorded pre-irradiation are in green, and post-irradiation are in red. Also see Figure S7 for emission spectra recorded using λ_ex_ = 280 nm.

To further examine the chemical events unique to the 7F–^L^Trp-containing hVDAC2, we subjected the native proteins (without 7F–^L^Trp but with ^L^Trp incorporated; produced using *E. coli* grown in (i) Luria-Bertini (LB) media, and (ii) minimal media) to irradiation. Unexpectedly, we observed an increase in the fluorescence intensity for all the folded proteins at 430 nm (Figure 8). The end-state fluorescence spectra resembled post-irradiated hVDAC2 containing 7F–^L^Trp (Figure 9), while spectro-polarimetry confirmed no change in the secondary structure content of the samples (see Figures 4–5). These unusual findings suggested a possible UV-induced protein modification, which was independent of fluorine incorporation, but possibly involving tryptophan. Indeed, comparison across all variants with or without 7F–^L^Trp shows a pattern in the excitation and emission scans. hVDAC2–WT shows the highest intensity and is closely followed by W160 and W221. W75 and W86 show a lower intensity.

Next, to test if Trp residues were indeed involved, we produced Trp-less hVDAC2 (W0) both from LB, and minimal media with(out) 7-fluoro–indole. These folded proteins, when subjected to UV irradiation at 282 nm, also display an increase in the fluorescence intensity and show post-irradiation excitation and emission spectra similar to hVDAC2 carrying 7F–^L^Trp (see Figures 6–9, S5, S7), and with rates similar to W160 and W221 (Table 1). The results from W0, and comparison of the rate constants, allowed us to conclude that (i) in hVDAC2, tryptophan did not undergo photo-activated modification to generate the unusual fluorescence, (ii) the modification did not necessitate incorporation of an indole in a protein, and (iii) selectively positioned indoles can enhance the fluorophore formation.

In an attempt to study the modified protein, we extracted the post-irradiated samples with methanol. Adding methanol allows the protein to precipitate from the folding reaction, while also simultaneously extracting the DDM in the supernatant. We refolded the protein thus extracted in a freshly prepared folding reaction containing DDM. Surprisingly, the refolded protein did not exhibit the characteristic excitation/emission pair observed post-irradiation (data not shown). Therefore, we conclude that the addition of methanol reverses the modification. We obtained a similar reversal when chloroform was used for protein extraction. We additionally tested DDM samples containing mole equivalents of tryptophan (as amino acid) or 7-fluoro–indole (Figure S8), but did not obtain the unique fluorescence properties displayed by the protein. 7-Fluoro–indole shows an increase in fluorescence upon irradiation, associated with the removal of fluorine and return to canonical Trp-like behavior.

Overall, our results confirmed that while tryptophan may not undergo modification, its position in the barrel plays an important role in the determining the characteristics of the resultant fluorescent entity. Our finding that the modification is readily reversible and occurs also in W0 suggests an oxidative effect that can relate to another aryl group such as tyrosine.

### UV-induced photo-activated fluorophore formation is ubiquitous to other proteins

**Figure 10:**
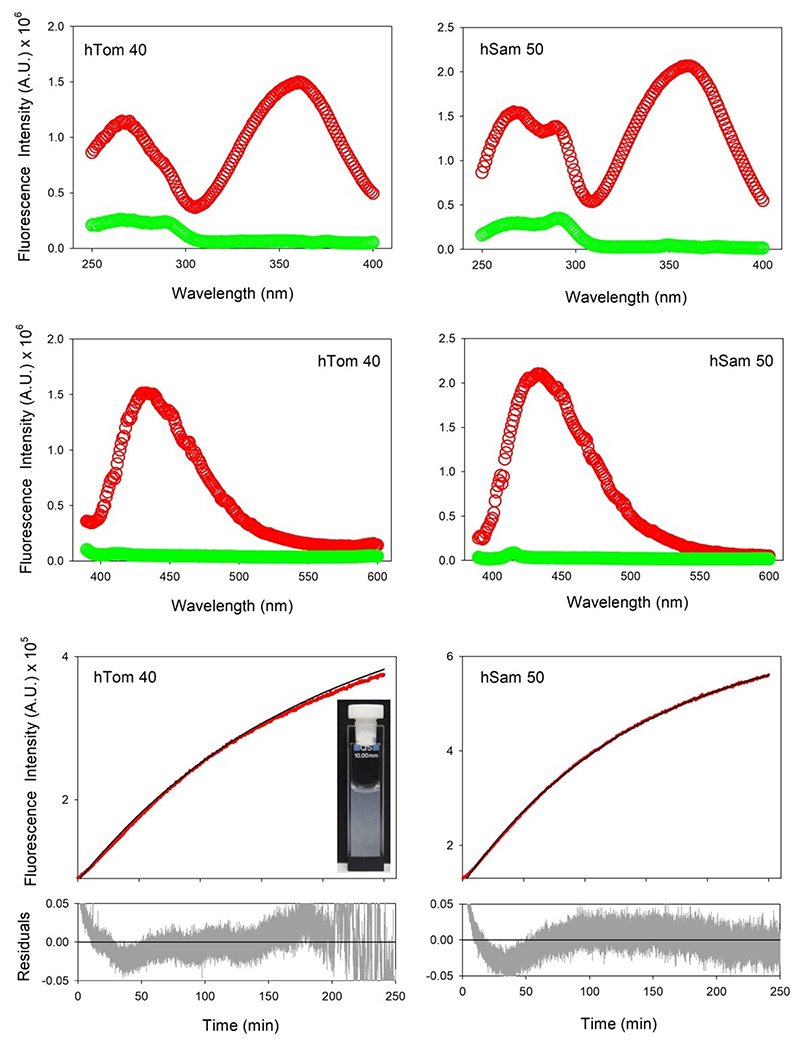
Kinetics of hTom40 and hSam50 cross-linking upon irradiation. UV-induced accumulation of cross-linked unusual fluorophore in human Tom40 (left) and human Sam50 (right). Interestingly, and unlike hVDAC2 and Sam50, hTom40 shows visible protein aggregation upon cross-linking (shown as inset).

Human Tom40 (hTom40) is a 361 residue 37.9 kDa transmembrane protein, which functions as the core protein of human mitochondrial translocase of the outer mitochondrial membrane (TOM) complex. hTom40 is synthesized in the cytosol, and is folded in the outer mitochondrial membrane through assistance from the mitochondrial sorting and assembly machinery (SAM).^[5a]^ In addition to sharing structural similarity with hVDAC2 (both are 19-stranded transmembrane β-barrels with short extramembranous helices at the N-terminus), human Tom40 also possesses three lipid-facing Trp residues that, upon protein folding, form an aromatic interaction network with 13 Phe, 9 Tyr, and other aliphatic side chains at the bilayer interface. To examine if the phenomenon was unique to hVDAC2, we subjected the transmembrane domain of hTom40 (lacking the 82-residue extramembranous N-terminal helix), to photo-activation. Notably, hTom40 also exhibited the red-shifted fluorescence (Figure 10), indicative of UV-mediated indole modification. Unlike hVDAC2, this modification destabilized the folded state of the protein, triggering protein aggregation (Figure 10). We also subjected the core SAM protein hSam50 (also a β-barrel protein), to the same conditions (Figure 10). hSam50 is a 16-stranded β-barrel, and additionally possesses 13 lipid-facing Trp residues, in addition to 29 Phe and 13 Tyr. Our results remained consistent even with a Trp-rich protein like hSam50, indicative of a UV-mediated modification that is not unique to hVDAC2. Furthermore, the rate of formation of the unusual fluorophore is comparable with the single-Trp hVDAC2 variants, and hTom40 (see Table 1), establishing that the process is independent of the number of indole rings.

**Figure 11:**
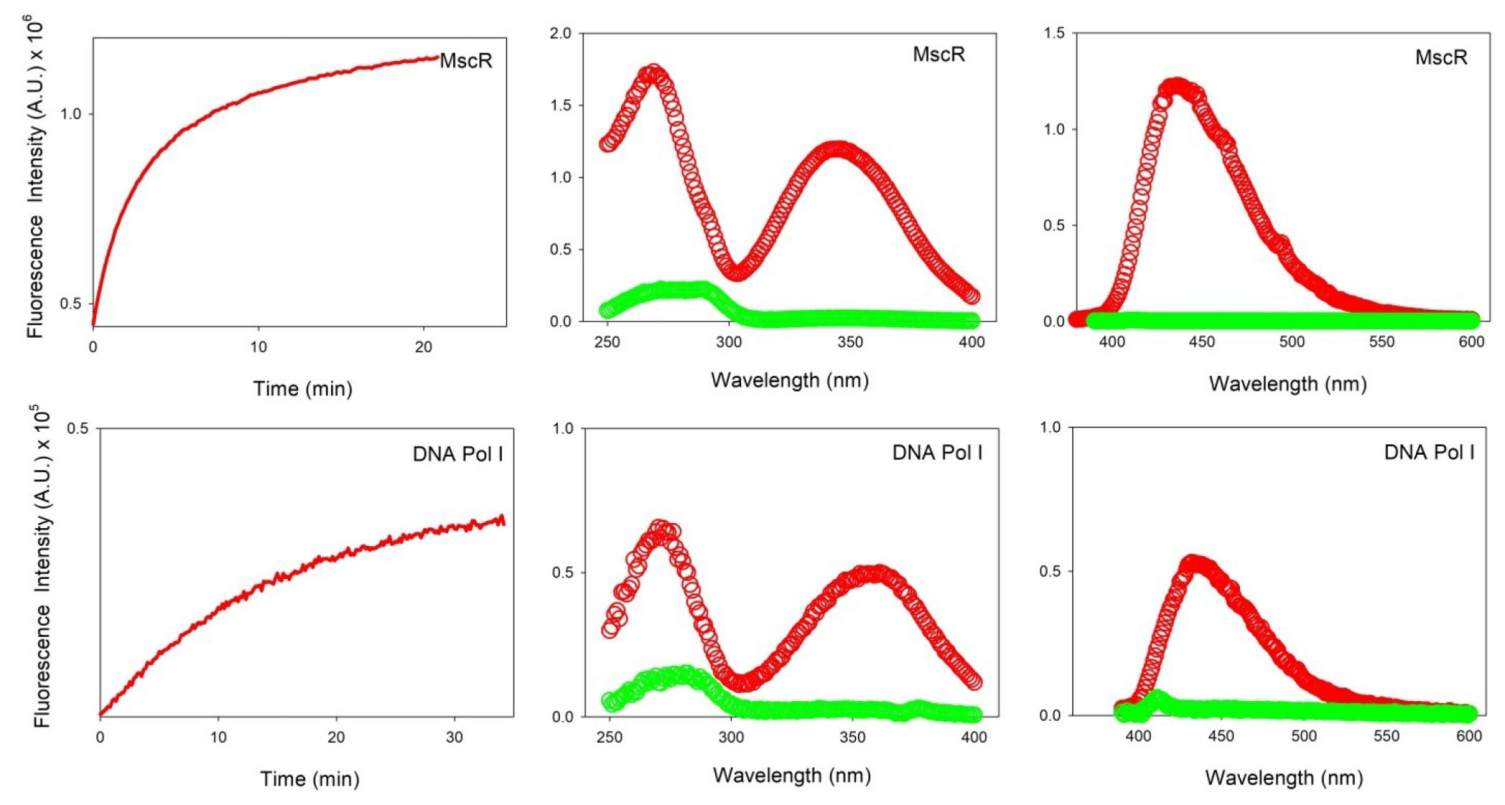
Soluble proteins *Mtb*–MscR and D29–PolI show accumulation of the unusual fluorophore were subjected to cross-linking at 282 nm. Kinetics of the reaction was monitored using emission at 430 nm. Also shown are the pre- (green) and post- (red) irradiation excitation and emission spectra. Excitation scans were recorded using a λ_em_=430 nm and emission spectra were recorded using λ_ex_ = 345 nm (MscR) and 360 nm (PolI).

Lastly, we examined whether a similar phenomenon can be observed with cytosolic proteins soluble in aqueous media. For this, we specifically chose two proteins from two different organisms, and with different number and positioning of tryptophan residues. The oxidoreductase S–nitrosomycothiol reductase (MscR)^[20]^ from *Mycobacterium smegmatis* is a 38.4 kDa single domain protein with 4 Trp, 14 Phe, and 4 Tyr. *Mtb*–MscR is a zinc-dependent dehydrogenase that detoxifies formaldehyde by converting it to formate.^[20a]^ Additionally, we used the DNA polymerase I (PolI) from the D29 virus (which infects *Mycobacterium tuberculosis*), which is a 68.0 kDa multi-domain protein with 10 Trp, 22 Phe, and 21 Tyr. PolI forms the vital entity for replication of the phage genome within the host bacterium.^[25]^ We subjected both proteins individually to the same conditions as the other proteins, and irradiated the samples at 282 nm. Both proteins show an exponential rise in fluorescence (rate constant = 14.4 h^−1^ for MscR and 3.6 h ^−1^ for polI) and red-shifted emission spectra post-irradiation (Figure 11). These results indicate that the modification is not unique to membrane protein systems. Although the final spectroscopic characteristics (λ_ex-max_, λ_em-max_, rates) might change with the change in hydrophobicity of the local environment, the modification itself will occur irrespective of whether it is a membrane or cytosolic protein. Indeed, MscR undergoes an extremely fast reaction to yield a modification with a slightly blue shifted λ_ex-max_= 345 nm, and the λ_em-max_= 430 nm similar to all other proteins studied here.

Overall, we find that the UV-induced production of the unusual fluorophore can occur in all proteins, and this can be enhanced by the presence of tryptophan residues in a position-specific manner. Since aromatic amino acids are indispensable for both the folding and stability of all proteins, mutating these residues will adversely affect the thermodynamics of the system and drive protein misfolding and aggregation. Indeed, even the conserved substitution of four tryptophans in the 294-residue hVDAC2 affects its structure (see Figure 2). Therefore, by combining previous findings on unusual protein modifications^[21,26]^ and our observations, we propose that our findings can be best explained as a UV-induced oxidation of the phenyl side chain of tyrosine. Furthermore, the lack of direct correlation between (i) aryl ring solvent accessibility, and (ii) number of tryptophans, with the rate of oxidation, suggests that the local environment of the tyrosyl ring determines the kinetics of the process. It remains to be seen if Phe additionally contributes to this unusual fluorophore, and whether further characterization -can be achieved with peptide-based models.

## Discussion

Proteins are indispensable workhorses for all essential biochemical function and regulation in every cell. For cell survival, protein turnover is a vital process, as it maintains an active population of proteins for cellular functions at all times. However, protein production is energy-intensive, which is why cells retain a mostly stable protein pool from its biogenesis to clearance by apoptosis.^[1]^ Despite high stability, proteins undergo undesired modifications by several mechanisms. These include modifications mediated by reactive oxygen and nitrogen species, which lead to irreversible loss-of-function and protein aggregation. Here, we show a rather unique modification occurring in a protein upon exposure to UV irradiation.

As shown in our study, the process of protein modification is slow, occurs even in the absence of metal catalysts or cofactors, and only requires prolonged exposure to UV. Hence, UV radiation, particularly for slow regenerating cells, can trigger the accumulation of oxidative modifications that adversely affect protein stability and function. For example, oxidative changes to the tyrosines can cause premature damage to corneal and retinal cells of the eye. Additionally, melanin synthesis in skin, which involves tyrosine, is closely associated with UV and visible radiation.^[27]^ UV-mediated photosensitized oxidation of tyrosine can lead to various modifications. Tyrosine modifications include cross-linking, hydroxylation, nitrosylation, nitration, and halogenation, based on the local environment of this amino acid, and can vary between soluble and membrane-associated proteins.^[21,26b,27–28]^ Consequently, tyrosines are often incorporated in proteins to buffer redox stress and scavenge oxidizing intermediates that can adversely affect active sites.^[29]^ Indeed, a recent finding identified how dityrosine cross-linking in human α-synuclein suppresses the aggregation dynamics of this protein.^[26h]^ Other unusual redox chromophores have also been characterized in proteins.^[26c–e,27–28,30]^

Thus far, direct evidence of aryl modification – in the absence of a secondary protein, small molecule, or metal ion as catalysts – giving rise to the unusual fluorescence we observe, has not been reported to our knowledge. A red-shifted emission spectrum in proteins with the closest match to our findings is with *diTyr* formation in calmodulin, when superoxide dismutase and divalent calcium are additionally present.^[26b]^ Our study used five different proteins, with(out) membrane lipid environments, different organisms as the protein source, buffer systems, and tests with(out) metal cofactors. Based on our findings from these diverse sample types, we conclude that cross-linking of tyrosine through photoradicals gives rise to the unusual fluorophore we observe in all systems.

Oxidative modifications and unusual cross-links are predominant in Trp-containing systems, and the propensity for the indole ring to form novel fluorophores is well documented.^[26f,g,29b,31]^ However, and despite their involvement in cross-linking,^[29b,32]^ reports of unusual fluorophores of *di*Tyr are relatively scarce than Trp. Tyrosine modifications can occur *in situ* in cells. It is as yet not known how the modified residues are restructured, so that the cells recover and protein function is restored in these cases. It is possible that enzymes and pathways exist to specifically recycle such modified proteins,^[29b]^ where these unusual bonds are reconfigured. Our ability to reverse this modification with methanol suggests the likely presence of a redox mechanism in cells that identify and repair protein damage due to UV-induced tyrosine oxidation. However, some oxidative modifications (such as those we find in human Tom40), can lead to protein aggregation. Failure to rescue damaged proteins, particularly under cellular stress or nutritional starvation,^[33]^ can trigger protein aggregation. The aggregation of essential proteins directly results in loss of protein function.^[27]^ This process then triggers premature cell death, or cause pathological accumulation of misfolded or non-functional proteins, and lead to cellular toxicity.^[34]^ Moreover, the oxidation-mediated aggregation can be particularly deleterious for neuronal development and can lead to neurodegenerative diseases.^[34a,35]^ The ability to reverse such modifications provides opportunities for developing suppressors of UV-induced oxidative build-up, and preventing the formation of toxic protein aggregates.

## Conclusions

Through incorporation of a singly fluorinated indole in human VDAC2, and our attempts to map the local dynamics and interface interactions in this vital human membrane protein, we serendipitously identified the formation of a photo-activated unusual fluorophore. We find that this oxidative modification involving tyrosines results in a substantially red-shifted fluorescence, which occurs in both membrane and soluble proteins irrespective of their structural characteristics and hydropathy, and is fully reversible. Mapping the precise modification through large-scale mutagenesis of tyrosines will result in protein destabilization. Therefore, further investigation of the chemical events underlying this specific photo-activated modification using alternative methods such as NMR, mass spectrometry,^[26g]^ and *in vivo* imaging, will open avenues to formulate therapeutics that suppress premature protein misfolding and aggregation, thereby prolonging cell viability.

## Figures and Tables

**Table 1:** Cross-linking rates of proteins when irradiated at 282 nm.

Protein	Rate constant Expt. 1 (h^−1^)	Rate constant Expt. 2 (h^−1^)	Mean (h^−1^)	Error (h^−1^)
With 7F–^L^Trp				
WT	0.7140	0.6060	0.6600	0.0540
W75	0.3180	0.3480	0.3330	0.0150
W86	0.5760	0.5700	0.5730	0.0030
W160	0.5940	0.6180	0.6060	0.0120
W221	0.3300	0.3060	0.3180	0.0120
W0	0.3060	0.3060	0.3060	0.0000
Without 7F–^L^Trp				
WT	1.4160	1.1040	1.2600	0.1560
W75	0.5100	0.9480	0.7290	0.2190
W86	0.2760	0.3480	0.3120	0.0360
W160	0.4680	0.3900	0.4290	0.0390
W221	0.4500	0.4680	0.4590	0.0090
W0	0.3180	0.3420	0.3300	0.0120
hTom40	0.3120	0.3360	0.3240	0.0120
hSam50	0.3240	0.4020	0.3630	0.0390

## Data Availability

The data that supports the findings of this study are available in the main text and supporting material of this article. All raw data files generated during the current study are available from the corresponding author upon request.

## References

[1] Baker RP, Urban S (2012). Nat Chem Biol.

[2] Calabrese AN, Radford SE (2018). Methods.

[3] Wimley WC (2003). Curr Opin Struct Biol.

[4] Walther DM, Rapaport D, Tommassen J (2009). Cell Mol Life Sci.

[5] Sayyed UMH, Mahalakshmi R (2022). J Biol Chem.

[6] Maurya SR, Mahalakshmi R (2016). FEBS J.

[7] Colombini M (2016). Biochim Biophys Acta.

[8] Tan W, Colombini M (2007). Biochim Biophys Acta.

[9] Khan A, Kuriachan G, Mahalakshmi R (2021). ACS Chem Neurosci.

[10] Martel C, Wang Z, Brenner C (2014). Mitochondrion.

[11] Rosenberg P (2022). Cell Calcium.

[12] Fukada K, Zhang F, Vien A, Cashman NR, Zhu H (2004). Mol Cell Proteomics.

[13] Jin K, Mao XO, Eshoo MW, del Rio G, Rao R, Chen D, Simon RP, Greenberg DA (2002). Neurochem Res.

[14] Jiang W, Du B, Chi Z, Ma L, Wang S, Zhang X, Wu W, Wang X, Xu G, Guo C (2007). J Neurosci Res.

[15] He Y, Wang W, Yang T, Thomas ER, Dai R, Li X (2022). Oxid Met.

[16] Yoo BC, Fountoulakis M, Cairns N, Lubec G (2001). Electrophoresis.

[17] Sirangelo I, Tavassi S, Martelli PL, Casadio R, Irace G (2000). Eur J Biochem.

[18] Lu M, Toptygin D, Xiang Y, Shi Y, Schwieters CD, Lipinski EC, Ahn J, Byeon IL, Gronenborn AM (2022). J Am Chem Soc.

[19] Maurya SR, Mahalakshmi R (2016). Biochim Biophys Acta.

[20] Wani SR, Jain V (2022). Protein Sci.

[21] Balasubramanian D, Kanwar R (2002). Mol Cell Biochem.

[22] Srivastava SR, Mahalakshmi R (2020). J Biol Chem.

[23] Mahalakshmi R, Franzin CM, Choi J, Marassi FM (2007). Biochim Biophys Acta.

[24] Srivastava SR, Zadafiya P, Mahalakshmi R (2018). Biophys J.

[25] Ford ME, Sarkis GJ, Belanger AE, Hendrix RW, Hatfull GF (1998). J Mol Biol.

[26] Aeschbach R, Amado R, Neukom H (1976). Biochim Biophys Acta.

[27] Recky Neyra JR, Serrano MP, Dantola ML, Lorente C (2021). Free Radical Biol Med.

[28] Malencik DA, Anderson SR (2003). Amino Acids.

[29] Gray HB, Winkler JR (2015). Proc Natl Acad Sci USA.

[30] Glover SD, Jorge C, Liang L, Valentine KG, Hammarstrom L, Tommos C (2014). J Am Chem Soc.

[31] Makwana KM, Mahalakshmi R (2015). Org Lett.

[32] Gatin A, Duchambon P, Rest GV, Billault I, Sicard-Roselli C (2022). Int J Mol Sci.

[33] Jain V, Kumar M, Chatterji D (2006). J Microbiol.

[34] Sarkar A, Rasheed MSU, Singh MP (2023). Antioxid Redox Signaling.

[35] Piccirillo S, Magi S, Preziuso A, Serfilippi T, Cerqueni G, Orciani M, Amoroso S, Lariccia V (2022). Antioxidants (Basel).

